# SAGES tool for assessing robotic surgery systems (STARSS)

**DOI:** 10.1007/s00464-025-11897-w

**Published:** 2025-07-17

**Authors:** Jackie S. Cha, Patrick Fuller, Matthew Ball, Sarah Samreen, Patricia Sylla, Dimitrios Stefanidis, The SAGES Robotic Committee

**Affiliations:** 1 Department of Industrial Engineering, Clemson University, Clemson, SC USA; 2 Department of Surgery, University of Texas Medical Branch, Galveston, TX USA; 3 Department of Surgery, Mount Sinai Health System, New York, NY USA; 4 Department of Surgery, Indiana University School of Medicine, 545 Emerson Hall, EH 411H, Indianapolis, IN 46202 USA; 5 Department of Industrial & Systems Engineering, University of Wisconsin - Madison, Madison, WI USA

**Keywords:** Robotic surgery, Assessment tool, Robotic system evaluation, Robotic-Assisted Surgery (RAS)

## Abstract

**Background:**

Robotic-assisted surgery (RAS) has transformed surgical practice, offering improved ergonomics, enhanced visualization, and improved dexterity. However, as new RAS technology and systems are developed, stakeholders including surgeons, hospital administrators, and regulators face challenges in evaluating system features and selecting appropriate platforms. Thus, the Society of American Gastrointestinal and Endoscopic Surgeons (SAGES) Robotics Committee aimed to develop a tool for assessing robotic surgery systems.

**Methods:**

Using a literature review and iterative feedback from SAGES robotic committee members, the relevant categories and corresponding specific metrics for evaluation of RAS systems were first defined. The final tool included categories and metrics agreed upon by > 80% of committee members. To determine the applicability and usability of this tool, the tool was applied to the evaluation of FDA-cleared robotic systems in the US and any needed changes were implemented.

**Results:**

The SAGES Tool for Assessing Robotic Surgery Systems (STARSS) that is a structured evaluation framework designed to facilitate the comparison of RAS systems was developed. The tool includes metrics spanning seven categories: General Information, Software Information, Hardware Information, Usability, Safety Features, Ergonomic Capabilities, and Training and Certification. Its application to Food and Drug Administration (FDA)-cleared RAS platforms is presented.

**Conclusions:**

STARSS enables systematic comparison of RAS systems by capturing technical capabilities, workflow integration, safety considerations, ergonomic support, and training infrastructure. It highlights both strengths and gaps in current platforms and can help guide clinical adoption, procurement decisions, research, and future system development.

During robotic-assisted surgery (RAS), the surgical team leverages robotic technology to complete minimally invasive surgery. RAS systems have been clinically used for the past two decades in a variety of surgical specialties such as general surgery and urology. In 1985, the first robotic system – the Programmable Universal Machine for Assembly 560 – was used in neurosurgery [1]; and almost a decade later, in 1994, the Automated Endoscope System for Optimal Positioning (AESOP) was approved by the Food and Drug Administration (FDA). Subsequently, the Zeus surgical robot was FDA approved in 2001 [2, 3], and these systems served as predicate devices to the da Vinci Surgical System (Intuitive Surgical) in 2003, which has become the leading RAS platform in the decades to follow [4].

Intuitive Surgical Systems have been the dominant robotic surgery platform in the market, with over 14 million procedures being completed since the initial launch of the da Vinci Surgical System [5]. This prominence of technology and associated profits have inspired other companies to develop RAS systems with similar designs and form factors to the da Vinci system. The typical components of RAS systems is composed of robotic arms, a surgical console (control), and a vision system [6]. These key features exist in many different fashions or types. For example, the Revo-i from Meerecompany and the Bitrack system from RobSurgical offer a similarly designed platform with a closed and open-faced console, respectively, single multi-arm patient cart, and visual cart set up [7]. Additionally, some platforms deviate from the size and multi-arm design of the da Vinci systems by offering smaller and modular single arm patient carts such as the Hugo RAS system from Medtronic [7].

The integration of RAS systems has highlighted strengths and opportunities in both the technology and its compatibility into the operating room (ORs), raising both critiques and praises about their impact on surgical care. Specific technical advancements such as the degrees of freedom, console design, and haptic feedback availability have been recommended [8]. Improvements to aid in the physical integration of these systems includes reducing the size or modularization of the robotic systems, as the systems are typically integrated into already compact and crowded ORs due to many other necessary pieces of equipment [9]. Furthermore, research on team member communication and workload with RAS system implementation is rising [10]. Studies have identified several characteristics of the RAS systems that both inhibit and enable effective team communication and workload such as the isolation of the surgical console making communication more difficult [11, 12]; inability to communicate through non-verbal communication due to teleoperation [13]; and the reduction of physical and mental workload of team members due to several technical upgrades [14]. Studies have reported mostly better ergonomic factors with RAS over laparoscopy, such as reduction of muscle activation across most upper-body muscle groups such as the triceps and deltoids with higher trapezius activation, and a reduction of self-reported fatigue associated with using the surgical console [15–17]. As different robotic platform designs have been introduced to address current design limitations, researchers have started to compare system components such as accessibility to the patient within the surgical field, the miniaturization of tools, and the modularity of the system to understand which systems may meet a specific adoption [7]. Moreover, with this technology adoption, wider, system-level considerations for implementation have been emerging such as the facilitation of robotic training programs, improvements for public awareness and understanding for certain demographics, and the development of federal guidance and regulation [18, 19].

As new iterations of robotic surgery technology and systems are developed, it is imperative for users (surgeons and other OR personnel), hospital administrators, and regulators to be able to compare RAS features, such as those described in the previous paragraph, to make informed decisions about their acquisition and use. Unfortunately, a reliable tool to evaluate RAS systems that considers all relevant aspects of RAS to surgeons and other stakeholders does currently not exist. Thus, the aim of this work was to develop a tool for the evaluation of surgical robotic systems regarding software, hardware, usability, ergonomics, and training characteristics. Such a tool could enable surgical staff, researchers, and manufacturers to determine the differences of each system and help standardize the information for evaluating and comparing future RAS systems.

## Materials and methods

This project was conducted by a working group of the Society of American Gastrointestinal and Endoscopic Surgeons (SAGES) Robotics Committee that included experienced robotic surgeons and human factors engineers. The first step was to define the relevant categories for evaluation of RAS systems and was accomplished via consultation of the literature and input from the SAGES robotic committee members. Each category was then populated with a list of specific metrics for evaluation based again on a literature review and input from committee members. The developed list of metrics under each category was then sent for review by committee members and modifications implemented based on their feedback. The final tool incorporated all relevant items of RAS systems that our committee members agreed upon (agreement > 80%). To determine the applicability and usability of this tool, working group members applied the newly developed tool to the evaluation of available robotic systems in the US that have undergone an FDA pathway to approval (e.g., clearance, approval, or granted) by consulting and reviewing RAS company user manuals and brochures and obtaining feedback from surgeon users. Any needed changes based on this evaluation were incorporated in the tool. The results of this evaluation and the final tool are presented in this paper.

## Results

Seven main categories were defined for this new RAS system evaluation tool (Table 1) including: General Information, Software Information, Hardware Information, Usability Information, Safety Features, Ergonomic Capabilities, and Training and Certification. A detailed description of the metrics, and their measurement units if applicable, within each category are described below. Table 1 shows examples of devices that have obtained FDA clearance, approval, or De Novo granted.

**Table 1:** Proposed assessment tool for RAS platforms, with example metrics completed for selected RAS systems/models

Device Name	Da Vinci Si	Da Vinci X^**^	Da Vinci Xi^**^	Da Vinci SP^†^	Da Vinci 5^***^	Senhance	Versius	Dexter Surgical Robot	Mira
General Information									
Manufacturer	Intuitive Surgical	Intuitive Surgical	Intuitive Surgical	Intuitive Surgical	Intuitive Surgical	Asensus Surgical	CMR Surgical	Distalmotion	Virtual Incision
Applications/ Procedures^*^	Urology, Gynecology, General Surgery, Cardiac, Thoracic [21]	Cardiac, Colorectal, General Surgery, Gynecology, Head and Neck, Lung Biopsy, Thoracic, Urology [22]	Cardiac, Colorectal, General Surgery, Gynecology, Head and Neck, Lung Biopsy, Thoracic, Urology [22]	Urology, Head and Neck [23]	General Surgery, Gynecology, Thoracic, Urology, Pediatric, Cardiac [24, 25]	General Surgery, Gynecology, Urology, Bariatric, Colorectal [26]	Urology, Gynecology, Colorectal, Upper GI, General Surgery [27]	General Surgery [28]	General Surgery [29]
Certification Status	FDA Clearance 2009 [30]	FDA Clearance 2017 [31]	FDA Clearance 2014 [32]	FDA Clearance 2014 [33]	FDA Clearance 2024 [34]	FDA Clearance 2017 [35]	FDA De Novo Granted 2024 [36]	FDA De Novo Granted 2024 [28]	FDA De Novo Granted 2024 [37]
System Cost	Between $0.5 million and $2.5 million [38]	Between $0.5 million and $2.5 million [38]	Between $0.5 million and $2.5 million [38]	Between $0.5 million and $2.5 million [38]	Unknown: estimated $2–2.5 million [39]	$1–1.2 million [40]	Approximate purchase price at $1.5 million [40]		
Market Status	No Longer Commercially Available	Commercially Available [41]	Commercially Available [41]	Commercially Available [41]	Limited Launch	Commercially Available [42]	Commercially Available [43]	Limited Launch	Limited Launch
Software Information									
Eye-Tracking	No	No	No	No	No	Yes [44]	No	No	No
Level of Automation	Level 1—Robot Assistance	Level 1—Robot Assistance	Level 1—Robot Assistance	Level 1 – Robot Assistance	Level 1 – Robot Assistance	Level 1—Robot Assistance	Level 1—Robot Assistance	Level 1 – Robot Assistance	Level 1 – Robot Assistance
Motion Scaling (Gain)	Yes [45]	Yes [46]	Yes [46]	Yes [47]	Yes	Yes [48]	Yes [49]		
Tremor Reduction	Yes [45]	Yes [46]	Yes [46]	Yes [47]	Yes	Yes [50]	Yes [49]	Yes [51]	
AI-embedding (image navigation guidance)	No	No	No	No	No	Yes [52]	No	No	
System-enabled Teleoperation	Limited	Limited	Limited	Limited	Limited	Limited	Limited	Limited	Limited
Hardware Information									
Patient Cart (Single/Multiple)	Single [53]	Single [46]	Single [46]	Single [54]	Single [25]	Multiple [44]	Multiple [55]	Multiple [56]	Single [57]
Size (Cubic Feet)	166.91 [21]	243.21 [46]	243.21 [46]	272.91 [54]	No official documentation for dimensions	Modular: No official documentation for dimensions	Modular: Console: 64.32 Bedside Unit: 7.27 [55]		24-inch length (Weighs 2 lbs) [57]
Surgeon Console (Open/Closed)	Closed [21]	Closed [46]	Closed [46]	Closed [54]	Closed [25]	Open/3d glasses [58]	Open/3d Glasses [55]	Open/3d glasses [59]	Open [57]
Surgeon Console (Sitting/Standing)	Seated [21]	Seated [46]	Seated [46]	Seated [54]	Seated [25]	Seated [58]	Both [55]	Both [51]	Both [57]
Dual/Assisting Console Availability	Dual console available [21]	Dual console available [46]	Dual console available [46]	Dual console available [54]	No official documentation	No [58]	No [55]		
Controller Type	Finger loops [21]	Finger Loops [46]	Finger Loops [46]	Finger Loops [54]	Finger Loops [60]	Laparoscopic Handles [58]	Hand Controllers [55]	Handle grips [57]	Handle Grips [57]
Camera Diameter (mm)	8.5 [21]	8 [46]	8 [46]	12 [61]	No official documentation	10 [62]	10 [55]	5–10 [57]	
Camera Quality	HD-3D [21]	HD-3D [46]	HD-3D [46]	HD-3D [63]	HD-3D [25]	HD-3D [44]	HD-3D [55]	HD-3D [57]	HD-3D [64]
Instrument Diameter (mm)	5, 8, 12 [21]	8 to 12 [65]	8 to 12 [65]	6 [61]	No official documentation likely compatible with Xi 8 to 12 [34]	3,10,5 [48]	5 [66]	10–12 [57]	
Instrument Degrees of Freedom	7 [67]	7 [68]	7 [68]	7 [61]	7 [25]	7 [69]	7 [70]	7 [57]	7 [57]
Instrument Reusability	Up to 10 times [71]	Up to 10 times [65]	Up to 10 times [65]	Up to 25 times [72]	No official documentation	unlimited [50]	13 times (projected) [49]	One time use [57]	Unlimited [57]
Incision Requirement (mm or size)	At least 5 mm [71]	At least 8 mm (Can be up to 12 mm for some accessory tools) [65]	At least 8 mm (Can be up to 12 mm for some accessory tools) [65]	25 mm [73]	No official documentation likely compatible with Xi 8 to 12 [34]	At least 3 up to 12 mm [74]	5 to 10 mm [75]	10 to 12 mm [57]	25 to 30 mm
Haptic Feedback	No	No	No	No	Yes	Yes [44]	Yes [43]		Yes [57]
Number of Robotic Arms / Ports	3/4 [21]	4/4 [46]	4/4 [46]	1/1 [54]	4/4 [25]	4/5 (Modular system, 1 per manipulator arm base) [48]	Up to 5/unknown (1 per bedside cart) [43]	3/5 [51]	Single Port (2 Arms) [64]
Boom Mounting Capabilities	No	No	No	No	No	No	No	No	No
Method of Entry	Transabdominal	Transabdominal	Transabdominal	Transabdominal	Transabdominal	Transabdominal	Transabdominal	Transabdominal	Transabdominal
Energy Ability (Radiofrequency/Monopolar/Bipolar Electrothermal Energy/Ultrasonic)	Monopolar, Bipolar, Radiofrequency, Ultrasonic [21, 76]	Monopolar, Bipolar, Radiofrequency, [65, 76]	Monopolar, Bipolar, Radiofrequency, Ultrasonic [65, 76]	Monopolar and Bipolar [72]	Monopolar, Bipolar, Radiofrequency, Ultrasonic	Monopolar, Bipolar, Ultrasonic [77]	Monopolar, Bipolar, Ultrasonic [62, 78]	Monopolar, Bipolar [57]	Monopolar, Bipolar [57]
Stapling Capability	Endowrist Stapler Compatible [79]	Endowrist Stapler Compatible [31]	Endowrist Stapler Compatible [31]	No	No official documentation likely compatible with Xi Endowrist stapler [34]	No	No	Open platform [51]	No
Perioperative Planning Capabilities	No	No	No	No	No	Yes [80]	No	No	No
Surgical Team Members required outside of console	Yes [21]	Yes [46]	Yes [46]	Yes [54]	Yes	Yes [58]	Yes [81]	Yes [51]	Yes [82]
Usability Information									
Spatial Needs (Size)	Large [7]	Large [7]	Large [7]	Large [7]	Large	Medium	Small [7]	Small [51]	Small [57]
Accessibility to Patient	Limited [7]	Limited [7]	Limited [7]	Limited [7]	Limited	Medium	Medium [7]	Good [57]	Good [57]
Representation of Environment (Fidelity)	Very Good [7]	Very Good [7]	Very Good [7]	Very Good [7]	Very Good	Very Good	Good [7]		
Modularity of Equipment	Medium [7]	Medium [7]	Medium [7]	Medium [7]	Medium	Good	Good [7]	Good [57]	Good [57]
Surgical Tool Miniaturization	Advanced [7]	Advanced [7]	Advanced [7]	Advanced [7]	Advanced	Advanced	Advanced [7]	Standard [57]	Good [83]
Compatibility with existing infrastructure	Good	Good	Good	Good	Good	Good [48]	No official documentation	Good [57]	Good [57]
Need for Laparoscopic Equipment	Yes	Yes	Yes	Yes	No official documentation	No official documentation	No official documentation	Uses Laparoscopic tools [57]	No [57]
Communication Rating	Limited [11]	Medium [11]	Medium [11]	Medium [11]	Medium [11]	Good [84]	Good [85]	Good [51]	Good [57]
Safety Features									
Speed of Undocking	Good [86]	Good [86]	Good [86]	Good [86]	Good [86]	Good [42]	No official documentation	Good [57]	Good [57]
Saliency of Alerts	No official documentation	No official documentation	No official documentation	No official documentation	No official documentation	No official documentation	No official documentation	No official documentation	No Official Documentation
Clinical Endpoints	Robotic platforms should be evaluated based on their FDA clinical endpoints that determine the overall effectiveness and safety of the medical device. These clinical endpoints include: 1) Operative Time; 2) Estimated Blood; 3) Loss; 4) Blood Transfusion Rate; 5) Conversion Rate; 6) Device-related Adverse Event Rate; 7) Intraoperative Adverse Rate; 8) Subjects with Major AE; 9) Subjects with Minor AE; 10) Length of Hospital Stay (Days); 11) Rate of Positive Surgical Margin; 12) Readmission Rate; 13) Reoperation Rate; 14) Mortality Rate								
Non-Clinical Bench Endpoints	Robotic platforms should be evaluated based on key FDA non-clinical bench studies that can evaluate the performance and safety of medical devices. These non-clinical bench studies include: 1) Biocompatibility/Materials; 2) Sterility/Reprocessing/Packaging/Shelf Life; 3) Electromagnetic compatibility/Electrical Safety; 4) Wireless Technology; 5) Software; 6) Cybersecurity; 7) Passing of non-clinical performance tests such as arm motion accuracy, instrument motion accuracy, system latency, droop rate, integrity, arm simulated use, system interfaces, electrical properties, and kit bench tests								
Ergonomic Capabilities									
Adjustable Console Features	Medium [87]	Medium [87]	Medium [87]	Medium [87]	High [60]	High [88]	High [89]	High [57]	
Fixed Chair or Interchangeable	Interchangeable [21]	Interchangeable [46]	Interchangeable [46]	Interchangeable [54]	Interchangeable [46]	Fixed [44]	Interchangeable [55]	Interchangeable [51]	
Sit or Stand or Both	Sitting [21]	Sitting [46]	Sitting [46]	Sitting [54]	Sitting [46]	Sitting [44]	Both [55]	Both [51]	
Viewer/Display Angle	40–60 degrees [87]	40–60 degrees [87]	40–60 degrees [87]	40–60 degrees [87]	40–60 degrees [87]	Open Console [90]	Open Console [90]	Open Console [51]	Open Console [57]
Foot Pedals Available	Yes [21]	Yes [46]	Yes [46]	Yes [54]	Yes [46]	Yes [44]	No [55]	Yes [51]	Yes [57]
System Ergonomics Assessment and Feedback	No	No	No	No	No	No	No	No	No
Training									
Simulation tools availability for skills training	Da Vinci skills simulator	Da Vinci skills simulator, Intuitive Learning online learning platform SimNow simulation platform Advanced Tissue Models Remote Case Observation Telementoring	Da Vinci skills simulator, Intuitive Learning online learning platform SimNow simulation platform Advanced Tissue Models Remote Case Observation Telementoring	Da Vinci skills simulator, Intuitive Learning online learning platform SimNow simulation platform Advanced Tissue Models Remote Case Observation Telementoring	Da Vinci skills simulator, Intuitive Learning online learning platform SimNow simulation platform Advanced Tissue Models Remote Case Observation Telementoring	Senhance Simulation	Versius Trainer	VirtaMed Simulator Didactic sessions Hands-on workshops [91]	
Trainers Available (Online/In-Person)	Online and In-person	my intuitive app, Case observation Online learning In-service training Simulation and skills training OR care team training Technology training On-site training Proctoring Advanced training Events Curriculum development	my intuitive app, Case observation Online learning In-service training Simulation and skills training OR care team training Technology training On-site training Proctoring Advanced training Events Curriculum development	my intuitive app, Case observation Online learning In-service training Simulation and skills training OR care team training Technology training On-site training Proctoring Advanced training Events Curriculum development	my intuitive app, Case observation Online learning In-service training Simulation and skills training OR care team training Technology training On-site training Proctoring Advanced training Events Curriculum development	Asensus Surgical Learning Academy, Online learning, On-site training, Proctoring, Surgical team training	CMR e-learning platform, Residential training course, On-site training, Surgical team training, In-person preceptors	Dexter Academy fundamentals training and e-learning, On-site training [91]	MIRA Fundamentals Training, Proctoring Program, and MIRA IQ[82]
Requirements for Proficiency	No official documentation	No official documentation	No official documentation	No official documentation	No official documentation	No official documentation	No official documentation	No official documentation	
Performance Enhancement Rating (e.g. Surgical Dexterity)	Good [92]	Good [92]	Good [92]	Good [92]	Good [92]	No official documentation	No official documentation	No official documentation	
Availability of Skills Curriculum	Yes [93]	Yes [93]	Yes [93]	Yes [93]	Yes [93]	Yes [94]	Yes [95]	Yes [91]	Yes [82]
Availability of training programs for OR team	Yes [96]	Yes [96]	Yes [96]	Yes [96]	Yes [96]	Yes [97]	Yes [98]	Yes [91]	Yes [82]
Availability of Assessment Tool/ Metric	Yes [93]	Yes [93]	Yes [93]	Yes [93]	Yes [93]	Yes [94]	Yes [94]		Yes [82]
Availability of Performance Data/Feedback	Yes [93]	Yes [93]	Yes [93]	Yes [93]	Yes [93]	Yes [94]	Yes [94]		Yes [82]
Availability of Training Support	Yes [96]	Yes [96]	Yes [96]	Yes [96]	Yes [93]	Yes [97]	Yes [98]	Yes [91]	Yes [82]

*Platforms are categorized by surgical procedures; if a platform can only be used in a specific general surgery procedure, then it will be categorized as “general surgery”

**The da Vinci X and Xi share the same instruments and technology apart from the patient cart, therefore user manuals and resources for the Xi were assumed to be identical for the da Vinci X

†FDA has cleared this system for urology and transoral otolaryngology procedures but is not indicated for use in general laparoscopic surgery

***Due to the novelty and limited availability of the da Vinci 5, some metrics are inferred based on available information for both the da Vinci 5 and da Vinci Xi due to the FDA’s determination that the device is substantially equivalent to the Xi model for some components [34]

Blank cells represent information not available to the authors at the time of writing this manuscript

### General information

This includes the device information that would distinguish it in a holistic landscape including the Device Name and Manufacturer. The applications and procedures provide the current indications for use, and the certification and market status give detailed information on the commercial availability and regulatory approval of the systems such as by the FDA. The system cost is also placed on the table for convenience; however, this may change due to both time and installation differences for each system, and prices can vary for the same system by geographic location as well. The market status of the device is also noted.

### Software information

This includes the software capabilities of each system. These metrics represent the capability of the device from a software perspective such as the amount of gain that translates to console manipulation, automation, etc. Binary variables include eye-tracking, motion scaling, and tremor reduction with a “Y” identifying if the system is operational with this feature and an “N” identifying if the feature is absent. The level of automation is based on the Levels of Autonomy in Surgical Robotics (LASR) taxonomy that assigns a score ranging between 0 (no autonomy) and 5 (fully autonomous) [20]. Artificial intelligence (AI)-embedding specifies any features that includes AI. Teleoperation defines the distance capabilities and teleoperation capabilities of the robotic system in terms of both delay time and connection types. This metric will be based on a qualitative scale adapted from Cepolina & Razzoli [7] (limited, medium, or good), with limited indicating feasible teleoperations being limited to the console being in the room of the surgical procedure and good indicating the ability for transatlantic or long-distance teleoperation. This metric of telepresence/ teleoperation refers to operating from a place that is physically distant from the operating room with the patient.

### Hardware information

The hardware information includes the equipment capabilities, compatibility, and contents that are used to perform the procedure. Information on each component of the robotic surgical device is contained within this section including the information on the patient cart or vision carts quantity; size of the robotic systems in cubic feet; the open or closed surgeon’s console; and the sit/stand capabilities of the surgeon’s console. Other surgeon console metrics include the controller type distinguishing devices that use fingertip controls, joysticks, laparoscopic tools, or other types. Specific tool metrics include camera diameter in millimeters, camera quality in definition, instrument (laparoscopic tools) diameter in millimeters, instrument degrees of freedom, instrument reusability (count), and incision size requirement in millimeters. Additional metrics include the presence of haptic feedback from the platform, the number of robotic arms, the presence of boom mounting capabilities, the number of ports, and if the system is endoluminal or transabdominal. Lastly, the energy availability (monopolar, bipolar, or both), stapling capability, perioperative planning capabilities, and the need for surgical team member assistance were also included.

### Usability information

Usability information includes metrics about the reported user experience of RAS systems. Usability metrics include compatibility with current surgical workflows [7] such as the spatial size of the RAS system relative to the surgical room (small, medium, large), the accessibility to the patient from the surgeon’s console (limited, medium, or good), representation of the operative field or the fidelity of the operative field to the surgical team (fair, good, very good), modularity or ability to adapt the equipment to the surgical operative field (limited, medium, good), and surgical tool miniaturization (standard or advanced). The integration of equipment is an important metric to compare robotic platforms in terms of their ability to integrate with operating room equipment (limited, medium, or good). Additionally, each platform is rated based on their need for laparoscopic equipment to prepare for the use of the RAS (yes or no). Finally, the communication metric is indicative of the team’s ability to effectively utilize verbal and non-verbal communication during surgery (limited, medium, or good).

### Safety features

The safety features category includes important safety metrics to ensure patient and clinician safety. Elements included in this category include the speed of undocking the robotic platform during an emergency (limited, medium, good), the saliency of any alerts from the robotic platform (limited, medium, good), and the safety of the robotic platform based on commonly used clinical endpoints and non-clinical bench endpoints as regulated by the Food and Drug Administration (FDA). These FDA standards should be assessed according to the regulatory guidelines listed both on the evaluation tool and specified FDA regulatory tests outlined in relevant regulatory documents.

### Ergonomic capabilities

The ergonomic capabilities are the general ergonomic, movement, or set-up metrics associated with each robotic system. This includes metrics for the adjustability of the surgeon console (limited, medium, or good), ability to swap out chairs, the capability of standing or sitting (for each surgical team member), the required neck bending (for both surgeon console and vision cart), and the foot pedal availabilities of the device (both surgeon console and vision cart). Lastly, whether the RAS platform offers automated ergonomic assessments or feedback on ergonomics can also be evaluated (limited, medium, or good). These metrics emphasize the ergonomic demands experienced by the surgical team when utilizing a RAS device.

### Training

The training category includes training availability for each system. This includes the availability of simulators for training purposes, the type of training – whether in-person or online. The learning curve metric is measured in procedures and should be the number of procedures to proficiency for a specific surgical task such as suture and knot tying. Next, dexterity is measured based on whether the RAS offers improved surgical performance such as dexterity enhancement when compared to performing the same task via laparoscopy (limited, medium, good). Finally, metrics for the availability of a skills curriculum for training, availability of training for the entire OR team, performance assessment tools or metrics, performance feedback, and the availability of training support were included as they are critical components for effective training in RAS. These metrics are here to provide general guidance on the levels of training available for each device as it is critical to have proficient surgical teams to complete procedures safely.

## Discussion

The proposed summative evaluation tool presented in this paper aims to outline the fundamental components of RAS systems and allow for comparisons across these systems for future research, surgical, and developmental needs. It is important to note that this tool is summative evaluation of RAS systems as they are released on the market to assist healthcare systems and other stakeholders in their decision-making process of choosing an RAS system and to serve as a competitive benchmarking along important metrics for manufacturers to consider during development and exploration. Each set of metrics aims to identify key aspects of these systems for the safe performance of RAS. Further, we ensured that all items would be applicable to new and advanced RAS systems as they were being developed and included elements that may not be currently available but were of importance to our surgeon stakeholders and likely will be included in the future. An example is the addition of proficiency requirements for training in each RAS platform. Such information is relevant to surgeons who want to adopt a new RAS system into their practice and may provide a good comparison of the ability of each system to enhance surgical performance. For example, if one RAS system enables expert performance on a task in half the time of another, it may indicate that the particular system may enhance surgeon ability better. It is important to note, however, that to enable comparisons among RAS systems, surgeon performance and learning curves should be assessed on the same task or procedure using the same expert performance levels. The prior development of the Fundamentals of Robotic Surgery curriculum and associated simulation model for assessment [99] may provide a great platform to accomplish this goal.

Our experience with using this tool to evaluate existing RAS systems provides some important insights that merit discussion. First, due to the longer presence and market dominance of the Da Vinci platforms (Intuitive Surgical), more information is readily available and more research has been done to expand its application to different surgical procedures [4]. This may provide the Da Vinci systems with an advantage over newer platforms due to it being a more well-documented and familiar system that is now applicable to a growing breadth of surgical procedures. However, the wider adoption and presence of information on Da Vinci platforms also contributes to a better understanding of any shortcomings and disadvantages of these platforms when compared to other RAS systems. Further, newer platforms may have been developed in an effort to address limitations of existing platforms. This abundance of information is juxtaposed with the smaller amount of information available with newer RAS platforms. This limited literature makes it difficult to identify many metrics of importance, especially if those metrics or components are not highlighted in brochures and user manuals provided by the company. This limited information and novelty with many other platforms also means that very few disadvantages or shortcomings could be reflected in the metrics table.

This tool will serve as a helpful resource for surgeons, prospective buyers, device manufacturers, and researchers by providing a standardized summary of important metrics to consider. Further, the metrics allow stakeholders to assess and compare the strengths and weaknesses of individual robotic platforms to make informed decisions on purchasing, areas for improvement, the identification of emerging trends, and opportunities for additional research and development.

In conclusion, the goal of this project was to develop an encompassing set of metrics for both current and future RAS systems. This tabular framework allows for direct comparisons of systems and helps identify the gaps of current RAS systems for future research opportunities. As new RAS systems are being developed, they should report on these metrics to ensure clear system qualities are communicated to the clinical specialists.
